# Analysis and Discrimination of Canadian Honey Using Quantitative NMR and Multivariate Statistical Methods

**DOI:** 10.3390/molecules28041656

**Published:** 2023-02-09

**Authors:** Ian W. Burton, Mohsen Kompany-Zareh, Sophie Haverstock, Jonathan Haché, Camilo F. Martinez-Farina, Peter D. Wentzell, Fabrice Berrué

**Affiliations:** 1 Aquatic and Crop Resource Development, National Research Council of Canada, Halifax, NS B3H 3Z1, Canada; 2 Trace Analysis Research Centre, Department of Chemistry, Dalhousie University, P.O. Box 15000, Halifax, NS B3H 4R2, Canada; 3 Canadian Food Inspection Agency, 1400 Merivale Rd, Ottawa, ON K1A 0Y9, Canada

**Keywords:** nuclear magnetic resonance (NMR), honey, quantitation, multivariate statistical analyses, food adulteration

## Abstract

To address the growing concern of honey adulteration in Canada and globally, a quantitative NMR method was developed to analyze 424 honey samples collected across Canada as part of two surveys in 2018 and 2019 led by the Canadian Food Inspection Agency. Based on a robust and reproducible methodology, NMR data were recorded in triplicate on a 700 MHz NMR spectrometer equipped with a cryoprobe, and the data analysis led to the identification and quantification of 33 compounds characteristic of the chemical composition of honey. The high proportion of Canadian honey in the library provided a unique opportunity to apply multivariate statistical methods including PCA, PLS-DA, and SIMCA in order to differentiate Canadian samples from the rest of the world. Through satisfactory model validation, both PLS-DA as a discriminant modeling technique and SIMCA as a class modeling method proved to be reliable at differentiating Canadian honey from a diverse set of honeys with various countries of origins and floral types. The replacement method of optimization was successfully applied for variable selection, and trigonelline, proline, and ethanol at a lower extent were identified as potential chemical markers for the discrimination of Canadian and non-Canadian honeys.

## 1. Introduction

Honey is a naturally sweet food obtained primarily from honey bees (Apis mellifera) consisting of a solution of mainly glucose and fructose at a concentration of about 80% in water. Honey is cultivated on a large scale around the world and different floral types from different geographical regions are recognized for their unique flavors and other properties such as medicinal benefits [1,2,3]. Approximately 2–3 percent of honey is small amounts of other compounds which are responsible for the specific organoleptic properties of specific honeys. These compounds include other di- and trisaccharides, amino acids, organic acids, and other small organic molecules. In fact, some honeys, for example New Zealand Manuka honey, are defined by a set of several unique chemical markers [4]. Other honeys may have chemical components that are unique to their geographical region or floral type [5].

The popularity of honey along with the limited supply of commercial honey raises the opportunity for adulteration [6]. Finding methods for detecting adulteration is important in assuring the quality of food products and ensuring the security of locally produced foods. This has been done previously using ^1^H NMR to identify adulteration of Robusta coffee in Arabica coffee and classify botanical extracts using chemical barcoding methods [7,8]. Stable isotope ratio mass spectrometry (SIR-MS) is the most common method for identifying adulteration in honey [9,10]. The ability of NMR to provide quantitative data for many chemical compounds in a single measurement suggests that 1D ^1^H qNMR spectroscopy coupled with statistical methods such as PCA or PLS-DA can be used as a reliable method for assuring honey security.

NMR spectroscopy is a powerful technique for the study of complex mixtures. The uniform molar response and the dispersion of signals at high magnetic fields allow the quantitative analysis of multiple chemical markers in a single spectrum with a vast dynamic range. This has been illustrated in the analysis of pure compounds many times in the literature [11,12,13]. However, as the number of compounds in a mixture increases, it becomes more difficult to extract quantitative information from the spectrum. In most cases, only a limited number of chemical markers can be identified and quantified. Multivariate statistical methods can be used in these cases to extract useful information from the spectra. This strategy has been previously illustrated in honey to classify honey based on geographical origin, floral type, and adulteration [14,15,16,17,18,19].

**Figure 1:**
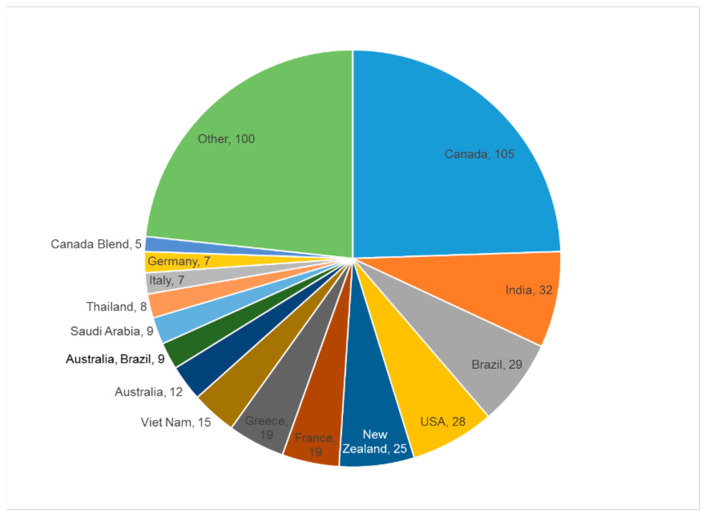
Geographical distribution of the honey samples.

To address the growing concern of honey adulteration in Canada and globally, the Canadian Food Inspection Agency conducted two campaigns in 2018 and 2019 to collect honey samples across Canada. Samples included honey and blends sourced along the supply chain including importers, brokers, blenders, domestic processing facilities, and retailers [20,21]. The results of the survey were published on the CFIA’s website, and it focused on the detection of C4 sugar adulteration using stable isotope ratio analysis (SIRA) and the addition of other C3 sugars by subcontracting these analyses to a third-party NMR analytical lab. As part of our collaboration with CFIA, a set of 424 honey samples was made available to further characterize their chemical content based on the NMR-based methodology described herein. In this library, honey samples declared as Canadian represent 25% of the library, followed by India, Brazil, the USA, and New Zealand as other countries of origin significantly contributing to the library (Figure 1). In addition to reporting the chemical composition of 424 honey samples using qNMR, the large number of Canadian samples allowed us to apply multivariate statistical methods in order to highlight trends and chemical markers to differentiate Canadian honey from the rest of the world.

## 2. Results and Discussion

Quantitative NMR has shown to be an analytical technique of choice for the characterization of the chemical composition of complex mixtures and food products [7,22]. The uniform molar response and the dispersion of NMR signals at high magnetic fields allow the quantitative analysis of multiple chemical markers in a single spectrum with a vast dynamic range of concentration. In this study, 33 honey components were successfully quantitated in 424 samples originating from more than 50 countries. The honey samples were dissolved in a 50 mM phosphate buffer solution to ensure repeatable chemical shifts for each compound, and in particular between the complex honey mixtures and the pure standards used for the annotation of the NMR signals. The 50 mM concentration was also found to be sufficient to compensate for variable amounts of organic acids in honey without losing much sensitivity in the NMR cryoprobe due to conductivity from the salt concentration [23]. A comparison of the NMR spectra recorded in both D_2_O and the buffered D_2_O solution (pH 7.4) is displayed in the supporting information (Figure S1). Dissolving the honey samples at pH 7.4 maintains the organic acids well above their pK_a_, and constitutes a particularity of the reported methodology herein when compared to literature data. For other protocols, an additional titration step at a pH of 4.5 was included, which could become a source of variation in large NMR data sets [24].

### 2.1. Quantitation of 33 Honey Components

**Figure 2:**
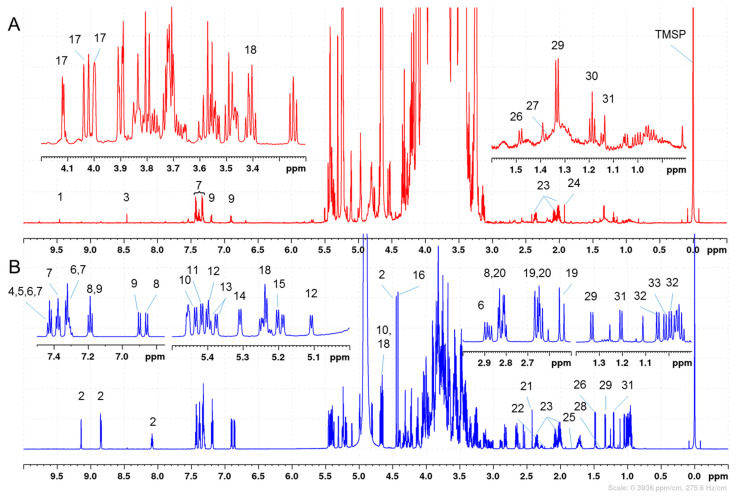
Assignments of the resonances used for the quantitation of the 33 honey components. (**A**) NMR spectrum of a Canadian honey sample. (**B**) Overlaid spectra of standards to support the annotation of the NMR signals. 5-HMF (1), Trigonelline (2), Formate (3), Leptosperin (4), Methyl syringate (5), Phenyllactate (PLA) (6), Phenylalanine (7), p-Hydroxyphenyllactate (HPLA) (8), Tyrosine (9), Maltose (10), Sucrose (11), Kojibiose (12), Nigerose (13), Turanose (14), Trehalose (15), dihydroxyacetone (DHA) (16), Fructose (17), Glucose (18), Citrate (19), Malate (20), Succinate (21), Methylglyoxal monohydrate (MGMH) (22), Proline (23), Acetate (24), proto-quercitol (25), Alanine (26), Lactate (27), Methylglyoxal dehydrate (MGDH) (28), Threonine (29), Ethanol (30), 3-Butanediol (31), Valine (32), Isoleucine (33), and TMSP (0.0 ppm).

NMR data for all 424 samples were recorded in triplicate and the concentrations of the 33 honey components were calculated by integrating individual and targeted NMR signals and averaging the results. The integration values were normalized by the number of protons and the concentrations were expressed either in g per 100 g of honey (g/100 g) for the major components or mg/kg. In addition to the unimolar response of NMR, each molecule in solution exhibits NMR signals corresponding to the number of protons borne on its chemical structures, and consequently add complexity in the NMR spectra but also redundancies for the quantitation of the targeted metabolites. However, many of these NMR signals are convoluted by overlapping resonances from other components, and Figure 2 represents the annotated resonances selected for the quantitation of the 33 compounds. It should be noted that no deconvolution was applied in this study.

The chemical components of honey includes the two major sugars fructose (17) and glucose (18); the carbohydrates maltose (10), sucrose (11), kojibiose (12), nigerose (13), turanose (14), and trehalose (15); the organic acids formate (3), citrate (19), malate (20), succinate (21), acetate (24), and lactate (27); the amino acids phenylalanine (7), tyrosine (9), proline (23), alanine (26), threonine (29), valine (32), isoleucine (33); and the non-standard amino acids p-hydroxyphenyllactic acid (HPLA) (8) and phenyllactate (PLA) (6). The other organic compounds are either attributable to specific floral origins or degradation products and include 5-hydroxymethylfurfural (5-HMF) (1), trigonelline (2), leptosperin (4), methyl syringate (5), dihydroxyacetone (DHA) (16), methylglyoxal monohydrate (MGMH) (22), methylglyoxal dehydrate (MGDH) (28), the deoxyinositol proto-quercitol (25), ethanol (30), and 1,3-butanediol (31). For example, DHA, leptosperin, methyl syringate, methylglyoxal, PLA, and HPLA are well-established chemical markers predominantly found in New Zealand Manuka honey. All the assignments were confirmed by recording NMR data on commercially available standards under the same conditions or comparing the ^1^H chemical shifts with previously reported literature data in the case of leptosperin, ethanol, formic acid, MGMH, MGDH, and acetic acid [4,25,26,27].

The minor sugars (turanose, kojibiose, maltose, sucrose, and trehalose) were quantified using the NMR regions of the alpha anomeric (H-1α) resonances between 5.15 and 5.55 ppm. The other resonances of these sugars were either too close to the non-quantitative water presaturation region or were overlapping with the NMR signals from fructose and glucose, the two major honey components which could represent up to 80% of the composition of honey [4]. In that case, the H-1α regions account for only a portion of the sugar diastereomers and the final concentration of sugars had to be calculated back by summing the integration values obtained for the total number of diastereomers or using the α/β anomeric ratios, accordingly. The assignments of anomeric protons and their ratios were determined based on the NMR spectra recorded for the pure standards under the same phosphate buffer condition. The anomeric ratios for each resonance were measured and the scaling factors are indicated in Table S1 in the supporting information. Since the glucose anomeric resonances were attenuated by the solvent presaturation, the overlapping protons H-4α and H-4β at 3.41 ppm (18 in Figure 2) were selected to report the concentration in glucose. These two NMR signals are distinct and do not overlap with any of the fructose ^1^H signals, and the contributions of other minor sugars were hypothesized to be minimal due to the lower order of magnitude differences in the concentrations. In a D_2_O solution, free fructose has a complex ^1^H NMR spectrum and is mainly found in three tautomeric forms, β-pyranose (68.2%), β-furanose (22.3%), and α-furanose (6.3%), with α-pyranose (2.7%) and keto tautomers accounting for approximately 3.2% [28]. As a result, the fructose concentration was estimated by summing three NMR regions counting for 2 protons: 4.098–4.130 ppm (β-fur H3, β-fur H4, α-fur H3), 4.013–4.05 ppm (β-pyr H6), and 3.985–4.013 ppm (β-pyr H5, α-fur H4). The integration regions and chemical shift assignments are reported in Table S1.

**Figure 3:**
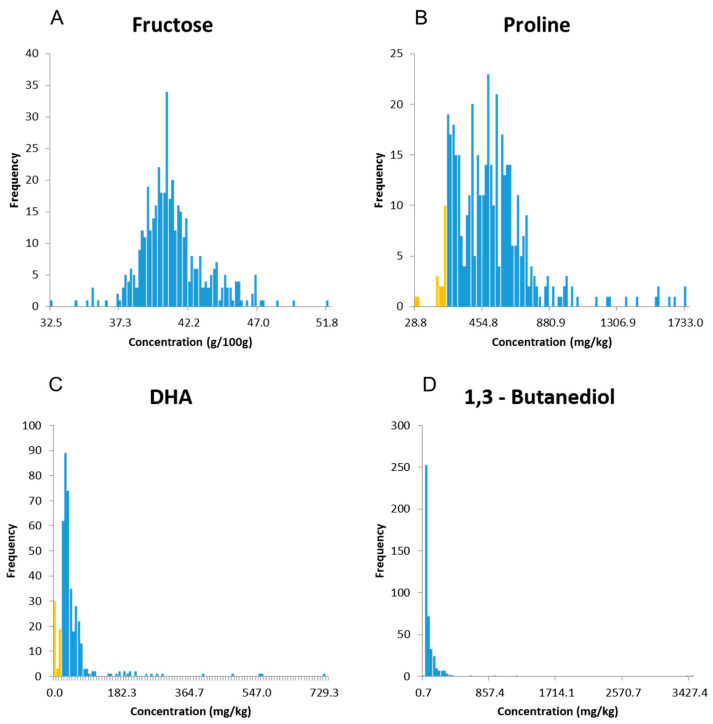
Frequency distributions of (**A**) fructose, (**B**) proline, (**C**) DHA, and (**D**) 1,3 butanediol by concentrations and across the entire library of honey samples. Values below the limit of quantitation (LOQ) are highlighted in yellow and concentrations above the LOQ in blue.

The quantitation results for all samples and honey components are quite extensive and the entire data set is provided in the supporting information in a tabulated format. The distribution of the concentrations observed across the data set varied for each analyte, and two approaches were selected to illustrate the range of concentrations observed for each component across the entire honey sample library. In the supporting information, Table S2 reports the average concentration of each analyte over all 424 samples and its standard deviation. The large standard deviation can be explained by the biological diversity of the honey samples composing the library including country of origin and floral varieties. In the manuscript, the frequency distribution histograms in Figure 3 display similar results in which the range of concentrations from lowest to the highest values were segmented into 1% bin size, and the Y-axis displays the count of honey samples with concentration values within the range of concentrations defined for each bin. A complete set of frequency distributions for all chemical markers is included in the supplementary information (Figure S2).

The frequency distribution obtained for the fructose concentrations showed a pattern similar to a Gaussian distribution with 32.5 and 51.8 g/100 g being the lowest and highest concentrations, respectively (Figure 3A). The amount of glucose and fructose are important markers for the authentication of honey and unusually high ratios are indicative of atypical honey or fraudulent practices [15].

Based on Figure 3B, the content of proline in honey has a wider distribution and is mostly influenced by the floras and the environments that bees were exposed to. The lowest concentration was as low as 28.8 mg/kg while the highest concentration was observed in a honey sample from Greece declared as “honeydew and thyme” with a value of 1733 mg/kg. When reporting concentrations, it is important to determine the limit of quantification (LOQ) for each analyte, which is the lowest concentration from which the amount of compound can be determined with accuracy, precision, and uncertainty. This value is well above the limit of detection (LOD) or the minimal concentration required to integrate an NMR signal above its noise level. The limit of quantitation was determined as the molar concentration of a singlet of 1 proton in a well-shimmed sample that would give a peak intensity with a signal-to-noise of 10:1. For the configuration of the 700 MHz NMR spectrometer, this LOQ was set at 0.01 mM and adjusted for each analyte based on the number of protons, the multiplicity of NMR signals, and the presence of eventual interfering resonances. To visualize the limit of quantitation, concentrations below the LOQ were highlighted in yellow and it was found to be at 230 mg/kg for proline.

As another example, dihydroxyacetone (16) is one of the key chemical markers associated with Manuka honey from New Zealand, and consequently, it is reflected in the frequency distribution in Figure 3C [4]. As expected, high concentrations of DHA were found in samples declared as New Zealand Manuka in the library with the highest concentration measured at 729 mg/kg, while 339 of the 425 honey samples exhibited concentrations below 87 mg/kg (the first bin size). Similarly, 420 of the 425 honey samples showed 1,3 butanediol concentrations below 378 mg/kg. However, two Taiwanese honey samples extended the concentration scale extensively with unusually high concentrations at 3427 and 3390 mg/kg as well as significant amounts of ethanol (246 and 243 nm/kg, respectively) and lower amounts of the other honey components overall. These observations represent only a few examples of how the NMR data set can be mined.

### 2.2. Multivariate Statistical Results

In this study, 105 samples were declared as Canadian while 172 of the 425 samples had a floral description associated with the honey samples. This provided a unique NMR data set to apply multivariate statistical methods and correlate chemical compositions, geographical locations, and floral types. The classification or differentiation of honey based on these features has been previously described in the literature [15,17,19,29]; however, to the best of our knowledge, this study represents the first example of using PCA, PLS-DA, and SIMCA methodologies to differentiate Canadian honeys from the rest of the world and identify putative chemical markers or trends.

#### 2.2.1. Principal Component Analysis

Principal component analysis (PCA) as an unsupervised cluster analysis technique is a common first step in data analysis. In absence of any previous information about classes, PCA visualizes the data in a reduced dimensional space based on the differences between samples in the data space with reduced dimensions. To have comparable variation in the concentrations of different metabolites in the data matrix from ^1^HNMR spectral peak areas, data columns were scaled by dividing by their standard deviations. PCA was then applied to the scaled concentration data from all 424 honey samples. Score plots from applying PCA on the scaled data did not show any discrimination between Canadian and non-Canadian samples. PC1 described 93.96%, while PC2 described 1.27% of the total variability which shows the high similarity of honeys to each other.

#### 2.2.2. PLS-DA on all Variables

The PLS-DA method is a type of standard PLS regression in which a y vector is generated by assigning a value of 0 or 1 to each sample, according to its class category. Predicted values by the model are related to either 0 or 1 based on a threshold. The true and predicted class memberships are then compared to evaluate how successful the model is at classifying the given samples.

In this report, honey samples are from many different origins (countries and florals) and metabolite concentrations are estimated from their NMR spectra. Based on these concepts, PLS-DA models were built using a training set that randomly selected 300 spectra out of 424 Canadian and non-Canadian samples. These samples were the same samples analyzed using PCA.

**Figure 4:**
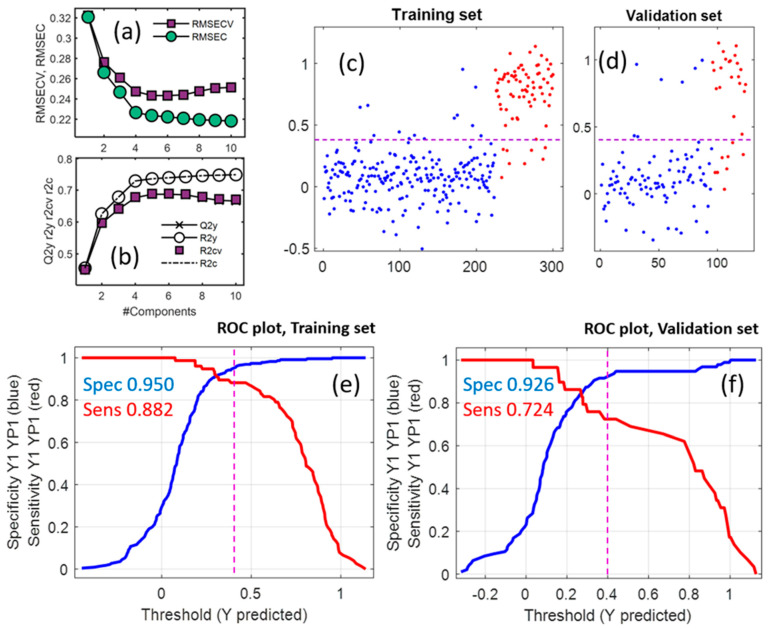
Results from applying the PLS-DA model on the data including all variables. RMSECV and RMSEC values (**a**) in addition to Q^2^ and R^2^ (**b**) values as the function of the number of latent variables were estimated using cross-validation. Estimated class membership (y) versus the sample number for training (**c**) and validation (**d**) sets (blue dots are truly non-Canadian and red dots are truly Canadian samples); ROC plots from the prediction of training (**e**) and validation (**f**) samples.

Cross-validation was used to select the optimal complexity of the PLS-DA model, which resulted in four or five latent variables considering minimum RMSECV values in different runs. Statistics indicated that the developed model could be acceptable to classify new samples. To validate the PLS-DA model and determine the proper number of latent variables a contiguous block cross-validation for seven subsets [30] was applied to the 300 samples of the training set. The resulting RMSECV and RMSEC from applying cross-validation using different numbers of factors are in Figure 4a. The minimum RMSECV value of 0.243 was obtained when the optimum number of latent variables was used, which was five. The RMSEC value when using five components in the PLS-DA model was 0.227. The maximum value of Q2 or R2CV (0.678, Figure 4b) was also obtained at the optimum number of variables.

Figure 4 includes the predicted class membership (y) for the honey samples in the training (c) and validation (d) sets and their classification as Canadian (red dots) and non-Canadian (blue dots). For the Canadian honeys, 67 out of 76 (sensitivity of 88.2%) samples in the training set and 21 out of 29 (sensitivity equal to 72.4%) samples in the validation set were correctly classified (as true positive). In the case of non-Canadian honeys, 213 from 300 samples (specificity = 0.950) in the training set and 88 from 124 samples (specificity = 0.926) were correctly classified (as true negative). As it is shown in the figure, estimated values of sensitivity and specificity were based on the threshold value of 0.4.

In the case of Canadian honeys, 11.8% of samples in the training and 27.6% in the validation sets were misclassified as false negatives (FNs). Incorrectly classified percentages of non-Canadian samples (false positive, FP) were 5.0% and 7.4% for the training and validation sets, respectively. ROC plots from PLS-DA on all variables for training and validation sets are illustrated in Figure 4e,f. Values of sensitivity (Sens) and specificity (Spec) as a function of threshold value are shown for training and validation sets. Sensitivity is the true positive rate and specificity is the false positive rate in class modeling. The applied threshold value for validation in this report is the same as that for training and the optimum value of the threshold is where Spec and Sens are high and comparable. The estimated values of Sens and Spec for the validation set are lower than those from the training set, although they are comparable. ROC results are summarized in Table 1.

#### 2.2.3. PLS-DA on Selected Variables

In this part, to reduce the complexity of the model by reducing the number of components (metabolites) from the 424 (honey samples) × 33 (components) concentration matrix, PLS-DA modeling was combined with RM-based variable selection. Based on a cross-validation procedure, discrimination of 104 Canadian honey samples from the 320 non-Canadian samples was performed. Like the previous section, data were randomly divided into training (300 samples) and validation (124 samples) groups, and a proper set of three variables was selected: trigonelline (2), proline (23), and ethanol (30). From running the RM variable selection PLS-DA procedure ten times, using randomized training and validation sets, the same set of three variables was selected and the performances of the models were similar to the PLS-DA model on all variables (Table 1). Performances of the models estimated from one or two selected variables were significantly lower than the model from all variables. Using three selected variables, true positive rates (Sens) of 86.8% for of training set and 78.4% for the validation set were estimated from the PLS-DA model. The true negative rate (Spec) from the model from the three selected variables for the training set was 95.3% and 95.4% for the validation set.

**Figure 5:**
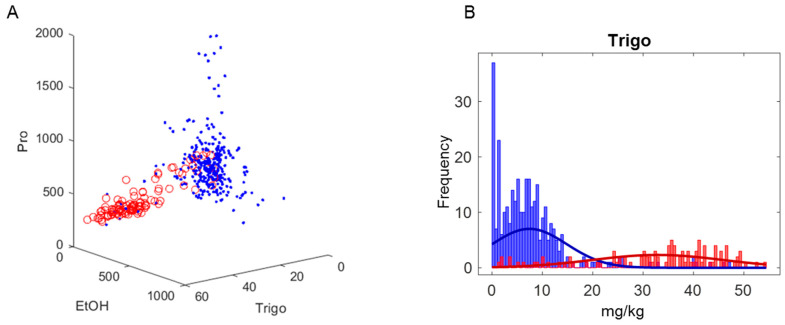
(**A**) Scatter plot of concentrations (mg/kg) of trigonelline (Trigo), proline (Pro), and ethanol (EtOH) in 424 honeys. The red circles are Canadian and the blue dots are non-Canadian honeys. All 3 scales are in mg/kg. (**B**) Distribution of trigonelline concentrations with Canadian samples labeled in red and all other samples in blue.

Concentrations (mg/kg) of three selected metabolites by RM-assisted PLS-DA discrimination are illustrated in Figure 5. In the 3D plot of concentrations of trigonelline (2), proline (23), and ethanol (30), Canadian samples (the red circles) are well separated from non-Canadian samples (blue dots). The most important variable for differentiation of the two classes is trigonelline, and samples containing higher than 20 mg/kg of trigonelline are primarily Canadian. Proline and ethanol concentrations are also remarkable in the discrimination of considered classes and ignoring them reduces the classification ability of the model.

**Figure 6:**
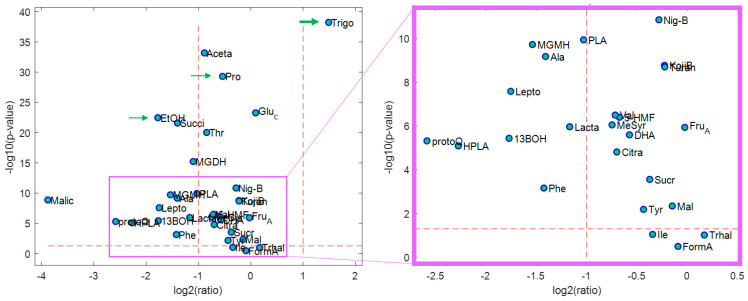
The volcano plot derived from fold change (ratio of mean values) and *t*-test (*p*-value) for univariate statistical analysis of the Canadian and non-Canadian honeys.

For further investigation of the specific differential compounds that took part in the intergroup separation, a volcano plot was drawn based on the results from fold change (ratio of means) and *t*-test (*p*-value) as the univariate statistical analysis. As shown in Figure 6, there are about 30 metabolites above the dashed line (*p* < 0.05), which means their concentrations are significantly different in the two classes. Metabolites with *p* < 0.05 include sugars such as maltose, sucrose, kojibiose, nigerose, turanose, fructose, and glucose in addition to amino acids such as proline, threonine, alanine, valine, phenylalanine, and tyrosine. Only trehalose (Trhal) has a *p*-value higher than 0.05 among the sugars, while isoleucine (Ile) is the only amino acid with a *p*-value higher than 0.05.

Alanine and phenylalanine are amino acids that have fold change values lower than 0.5 and their average concentrations are lower in Canadian honeys. Trigonelline is an alkaloid and the only metabolite with an average concentration of more than twice in Canadian samples compared to the others. Average concentrations of ethanol (EtOH), succinic acid, methyl glyoxal dihydrate (MGDH), methylglyoxal monohydrate (MGMH), leptosperin, hydroxyphenyl lactic acid (HPLA), 1,3 butanediol (13BOH), and lactate in Canadian samples are less than half of their average concentrations in non-Canadian samples. 5-hydroxymethylfurfural (HMF) often arises from the heating or storage of honey and has a lower content in Canadian honeys. Finally, the three selected metabolites by the model (Trigo, Pro, and EtOH) show *p* < 0.05 and different concentration ratios in Canadian and non-Canadian honeys, and Trigo can be considered as a potential marker for the discrimination between Canadian and non-Canadian honey.

#### 2.2.4. SIMCA Results

SIMCA, a class-modeling technique, was used to classify the honey samples coming from Canada and many different geographical origins. Class-modeling techniques aim at looking for similarities occurring among samples of the same class and model each category separately. As before, SIMCA models were built using a training set that randomly selected 300 spectra out of 424 Canadian and non-Canadian honey samples.

**Figure 7:**
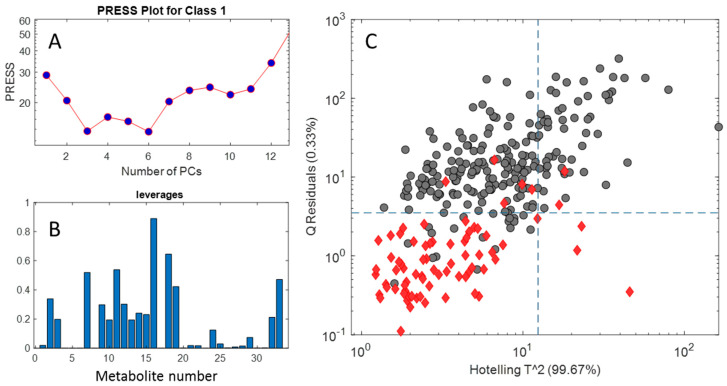
Results from SIMCA class modeling considering Canadian samples. Prediction error sum of squares (PRESS) plot from cross-validation of the Canadian model (**A**). Leverage plot of loadings related to each of 33 metabolites (**C**), and sample residuals versus sample leverages relevant to the Canadian class (**B**). In part (**B**), Canadian samples are the red lozenges and others are the gray circles.

The optimal complexity of the model, i.e., the number of PCs to be used to describe the class variability, was chosen on the basis of a CV procedure and the minimum estimated PRESS value indicated six PCs as the optimum for the class modeling (Figure 7A). Leverages of different chemical components estimated from the loadings of the SIMCA model are illustrated in Figure 7C. DHA (16), glucose (18), phenylalanine (7), sucrose (11), and trigonelline (2) are among the variables with high leverage in the PCA modeling of the Canadian class during SIMCA. Trigonelline is the common component between PLS-DA discriminant analysis and SIMCA class modeling.

The defined space area for the Canadian samples in the hyper-space of the model at a specified confidence level (95%) is illustrated in Figure 7B. Almost all of the non-Canadian samples (gray circles) are out of the class region of Canadian honeys regarding the significant residuals they show.

The sensitivity and specificity for the estimated model were estimated for both training and validation sets and similar results to that from PLS-DA were obtained. The geometric average between sensitivity and specificity in CV was selected as an optimality criterion so that the number of PCs was chosen as the one corresponding to the highest value of this figure of merit.

Classification results from the application of PLS-DA and SIMCA to the samples in training and prediction sets are shown in Table 1 as confusion matrices. In the SIMCA classification, eight out of sixty-eight Canadian honeys are misclassified in the non-Canadian group (FN) in the training set, and 6 of 37 in the validation set are FN.

In the PLS-DA, when using all variables (like SIMCA) 88.2% of Canadian samples are correctly classified in the Canadian group (TP) and 11.8% are misclassified (FN). Showing a higher value of TP (83.8% compared to 72.4%) and a lower value of FN (16.2 compared to 27.6) in the validation set, the prediction ability of the SIMCA model is better than the full variable PLS-DA.

Results from PLS-DA on the selected variables from the training set is 86.8% of correct classification of Canadians, which is comparable to 88.2% from the application of PLS-DA on all variables and SIMCA. In the case of the validation set, the TP percent of PLS-DA by variable selection is 78.4%, which is higher than that of the full variable PLS-DA.

Results show that the performance of PLS-DA is similar to that of the SIMCA method. However, the sensitivity of SIMCA in the validation set (83.8%) is higher than PLS-DA on full variables (72.4%) and the specificities from SIMCA for both training and validation sets are lower than that from PLS-DA. When applying PLS-DA to the three selected variables (trigonelline, ethanol, and proline) the performance does not change significantly, which means the simpler model with three variables contains the needed information for proper classification.

**Figure 8:**
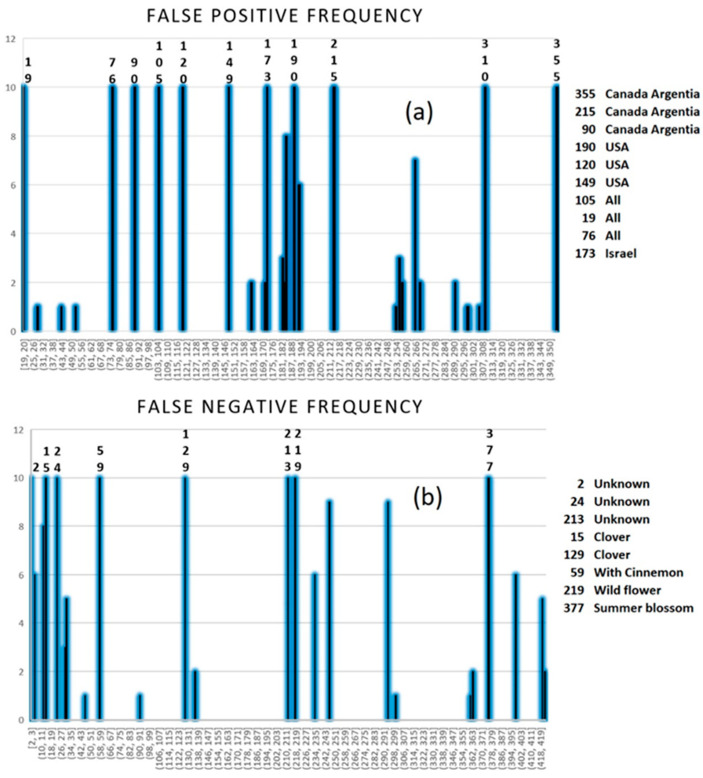
Frequency of false positive (**a**) and false negative (**b**) honey samples from running PLS-DA ten times on all components using four latent variables. Labels assign samples with maximum frequency.

Ten replicates of PLS-DA were applied to all metabolites using four latent variables (Figure 8). Ten non-Canadian samples were false positive (classified as Canadian) in all ten times running PLS-DA. There were three labeled as “Canada, Argentina” out of five Canadian mixtures, three USA samples, one “Israel”, and three labeled “All countries”. As the two pure Argentinian samples are TN, it seems possible that the major fraction of “Canada, Argentina” honeys are Canadian. Since Canada shares a large border with the US, it seems probable that honeys from the two countries may show similar properties and be classed together. Only one out of nine honeys from Israel is an FN.

Eight Canadian honeys were false negatives. From eight Canadian clover honeys, only two were repeatedly FN. Of six Canadian wildflowers, only one is FN. The summer blossom and the honey with cinnamon samples are FN. There are 73 Canadians with unknown floral origins and only three of them are classified as non-Canadian. The results of this study show that NMR spectroscopy coupled with multivariate methods holds the necessary information for the successful classification of Canadian and non-Canadian honey samples. Applying PLS-DA and SIMCA, as one-class classification models to predict honey samples, high classification rates were achieved. Application of the replacement method as a simple variable selection method reduced the number of applied variables in the model without loss of any information and revealed the metabolites with determining role in the separation of Canadian samples from non-Canadians using a set of 424 samples.

## 3. Materials and Methods

### 3.1. Sample Preparation

The set of 424 honey samples consisted of a few groups from unique countries with large amounts of samples along with a large number of diverse groups of countries and mixtures. For example, unique samples from Canada, the US, Brazil, India, Australia, and New Zealand made up 231 out of the 425 samples in just 6 classes. The rest of the samples make up 57 classes. The largest group of samples was identified as pure Canadian honeys consisting of 105 samples (25% of the total). In addition, there were 5 samples that had Canada listed as a component of a mixture of honeys. The large group identified as “Other” (100 samples) are from countries consisting of less than 5 samples or are blends of several different countries (Figure 1). The honey samples, contained in a 100 mL falcon tube, were heated from fridge temperature to 50 °C for one hour before mixing to ensure uniform sampling. Three 50 mg aliquots of each sample were accurately weighed and then dissolved in 1 mL of buffered D_2_O (50 mM NaH_2_PO_4_/Na_2_HPO_4_ buffered D_2_O at pH 7.4) containing 1.00 mM sodium trimethylsilylpropionate-d4 (TMSP). Samples were vortexed for 30 s and then 600 µL was transferred to 5 mm NMR tubes.

### 3.2. NMR Spectra

The ^1^D-^1^H NMR spectra were acquired on a Bruker Avance III 700 NMR spectrometer (Bruker Canada, Milton ON, Canada) operating at 700.15 MHz equipped with a 5 mm CPTCI cryogenically cooled probe. Samples were run at 20 °C using a 90-degree excitation pulse and presaturation of the residual HOD resonance during the relaxation delay of 11.88 s. The total duration between pulses was 15 s. Each sample was tuned and matched to 50 Ω resistive impedance and the 90-pulse duration was calibrated for each sample automatically using the standard nutation experiment. Spectra were acquired into 64 K complex points and averaged over 64 scans and 8 dummy scans.

The resulting spectra were processed with the Bruker Topspin software (v4.05, Bruker Canada, Milton ON, Canada). The spectra were Fourier transformed into 64 K real points after apodizing with a decaying exponential function with a line broadening factor of 0.3 Hz. The chemical shifts of all spectra were referenced to the methyl resonance of TMSP at 0.0 ppm. Spectra were phased to pure absorption and baseline corrected over regions using a 4th-order polynomial.

### 3.3. Peak areas and Concentrations

The spectra were integrated by importing the spectra into Octave and then taking the sum of the intensities of the points over the integration regions defined in Table S1. The concentrations of the chemical species were calculated using the methyl of the TMSP as an internal standard. The concentration of the internal standard was verified using external standard qNMR against triplicate samples of caffeine in D_2_O at a concentration of 4.09 mM using a method published previously [13]. The mean concentration of TMSP over all of the samples was 0.966 ± 0.05 mM, which was used as the concentration of the internal standard in the concentration calculations. Concentrations of the sugars are reported in g/100 g honey and all other chemical markers are reported as mg/kg honey, consistent with other studies. The complete table of concentrations can be found in the supplementary material.

### 3.4. Chemometrics Analyses

PCA as component modeling, in addition to PLS-DA [31,32,33] and SIMCA as discrimination analysis techniques, was applied to the concentration data estimated from ^1^H NMR peak areas after alignment, baseline correction, and other preprocessing. The goal was the discrimination of Canadian honey samples from other honeys. Honey samples were pure and mixtures from different countries around the world and some adulterated honeys were among the samples.

Before applying PCA, PLS-DA, and SIMCA (multivariate techniques), the concentration data matrix was scaled to standard deviations of columns. The concentration matrix was from 33 metabolites (columns) and 424 samples (rows), of size 424 × 33. PLS Toolbox version 8.2.1 (Eigenvector Research Inc., Wenatchee, WA, USA) [30] was used for data analysis.

The SIMCA and PLS-DA training sets were built from a random selection of 300 samples out of 425 honeys, and the validation set was the remaining 125 samples. SIMCA and PLS-DA methods were used to attain the one-class classification models for predicting the Canadian samples from other honeys.

#### 3.4.1. PLS

In the PLS-DA model, the optimum number of latent variables was chosen based on the predicted residual sum of squares (PRESS), which should be minimized, along with the Q2 and R2 values from regression. The prediction ability of the model was examined by computing the standard error of calibration (SEC) and standard error of validation (SEV). Contiguous cross-validation (the leave-five-in order-out procedure) was used to evaluate the performance of the model developed. For the PLS-DA model, three latent variables were selected for the separation of Canadian honey samples from others.

#### 3.4.2. SIMCA

Soft independent modeling of class analogy (SIMCA) [34] is a one-class classifier and among-class modeling technique [35]. In a one-class classifier model, instead of modeling several classes simultaneously, each group is considered using a separate model. A boundary is determined around each class and assigns a sample to that class if it falls within the boundary.

Considering all samples, a discriminant analysis classifier such as PLS-DA is used to separate samples into different classes, by determining the boundary between classes [31,36,37]. If there are two classes, the model is called a two-class classifier. For the SIMCA model, the number of principal components (PCs) used in each class model was determined using cross-validation and a 95% confidence level and 4 PCs were selected for the Canadian category.

#### 3.4.3. Replacement Method

The replacement method (RM) [38,39] of optimization is a useful grid search-based strategy to find the optimum and informative metabolites during a PLS-DA-based classification modeling. The replacement method (RM) [39] strategy has been successfully applied in QSAR/QSPR studies [40] and for variable selection in spectroscopy [41]. The main idea of the RM algorithm is to search for an optimal submatrix that includes a limited and fixed number of variables and results in minimum prediction error (RMSECV) in a multivariate regression model such as PLS. For starting the RM procedure, choose a subset of data with d variables at random, and perform regression and prediction. Replace one of the variables from the subset with each of the available variables keeping the best predicting set. One can start by replacing any of the d variables in the initial model, and a regression equation with d variables has d possible paths to the result. So, replace the entire remaining variables and repeat the whole process. At the end, we have the best model for the considered path. Proceed in the same way for all possible paths i = 1, 2, …, d, compare the resulting models, and keep the best one. The process will be repeated as many times as needed until the set of variables remains unchanged.

The RM generates models that are similar to genetic algorithms, although requiring a lower computation time [40]. RM can be preferable to the GA because the former takes into account the error in the regression coefficient and is not at random as the GA. In addition, the practical application of the GA requires the tuning of some parameters such as mutation probability, crossover probability, and generation gap, which is not the case in RM [42].

## 4. Conclusions

The present study illustrates that the high throughput NMR-based analysis of honey coupled with the multivariate analysis methods PLS-DA and SIMCA is a reliable and efficient way to analyze and differentiate honey from different geographical regions. The sample preparation method using buffered D_2_O at pH 7.4 was fast and easy and resulted in more reproducible NMR spectra than using an unbuffered solvent. Both PLS-DA as a discriminant modeling technique and SIMCA as a class modeling method proved to be reliable at differentiating Canadian honey from a large pool of honeys from various geographical regions through satisfactory model validation. The replacement method was successfully applied, and the selection of only three variables, trigonelline, ethanol, and proline, was found to be sufficient to separate Canadian samples from non-Canadian honey without loss of information. The ability to quickly differentiate Canadian honey from others is a key step to identify atypical Canadian honeys and subsequently trigger a more detailed chemical analysis to determine whether these outliers are the results of floral/geographical variations or putative fraudulent practices. Future work will focus on further refining the quantitative process using deconvolution in order to quantify additional chemical markers in honey. This study highlights that a simple and non-destructive sample preparation, in addition to recording high-quality ^1^H NMR data and using multivariate chemometric approaches, offers new analytical solutions to the industry and regulators for ensuring the authenticity and quality of honey products.

## Figures and Tables

**Table 1:** Confusion matrices from true and predicted classed by PLS-DA for all variables, PLS-DA on selected variables, and SIMCA models applied to the training and validation honey data sets.

**Training Set**	**Predicted Class**					
	**PLS-DA on All**		**PLS-DA on Selected**		**SIMCA**	
**True Class**	**Canadian**	**Non-Canadian**	**Canadian**	**Non-Canadian**	**Canadian**	**Non-Canadian**
Canadian	67 88.2%	9 11.8%	59 86.8%	9 13.2%	60 88.2%	8 11.8%
Non-Canadian	11 5.0%	213 95.0%	11 4.7%	221 95.3%	33 14.2%	199 85.8%
**Validation Set**	**Predicted Class**					
	**PLS-DA on All**		**PLS-DA on Selected**		**SIMCA**	
**True Class**	**Canadian**	**Non-Canadian**	**Canadian**	**Non-Canadian**	**Canadian**	**Non-Canadian**
Canadian	21 72.4%	8 27.6%	29 78.4%	8 21.6%	31 83.8%	6 16.2%
Non-Canadian	7 7.4%	88 92.6%	4 4.6%	83 95.4%	8 9.2%	79 90.8%

## Data Availability

The tabulated quantitative NMR results could be made available upon request to the corresponding author via e-mail.

## Supplementary Materials

The following supporting information can be downloaded at: https://www.mdpi.com/article/10.3390/molecules28041656/s1, Figure S1: Comparison of 25 honey samples on D_2_O vs. buffered D_2_O; Figure S2: Frequency distribution histograms for all analytes; Table S1: Integration regions and chemical shift assignments of chemical markers; Table S2: Average concentrations of each analyte over all 424 samples.

## Sample Availability

Samples of the compounds are not available from the authors.
