# Supplementary material for: Analysis and Discrimination of Canadian Honey Using Quantitative NMR and Multivariate Statistical Methods
Source: Molecules. 2023 Feb 9;28(4):1656. doi: 10.3390/molecules28041656 (PMC9959790; doi:10.3390/molecules28041656)
Supplement: Supplementary file 1 [file molecules-28-01656-s001.zip › molecules-2184855-supplementary.pdf]

Analysis and Discrimination of Canadian Honey using Quantitative NMR and Multivariate Statistical Methods.

Ian W Burton [1], Mohsen Kompany-Zareh [2], Sophie Haverstock [1], Jonathan Haché [3], Camilo Martinez-Farina [1], Peter Wentzell [2], and Fabrice Berrué [1,*]

[1] Aquatic and Crop Resource Development, National Research Council of Canada, Halifax, Nova Scotia B3H 3Z1, Canada
[2] Trace Analysis Research Centre, Department of Chemistry, Dalhousie University, Post Office Box 15000, Halifax, Nova Scotia B3H 4R2, Canada
[3] Canadian Food Inspection Agency, 1400 Merivale Rd, Ottawa, Ontario, K1A 0Y9, Canada
* Correspondence: fabrice.berrue@nrc-cnrc.gc.ca; Tel.: 902-402-3995

Figure S1: Comparison of 25 honey samples on D_2O vs buffered D_2O.

Table S1: Integration regions and chemical shift assignments of chemical markers.

Table S2: Average concentrations of each analyte over all 424 samples.

Figure S2: Frequency distribution histograms for all analytes.

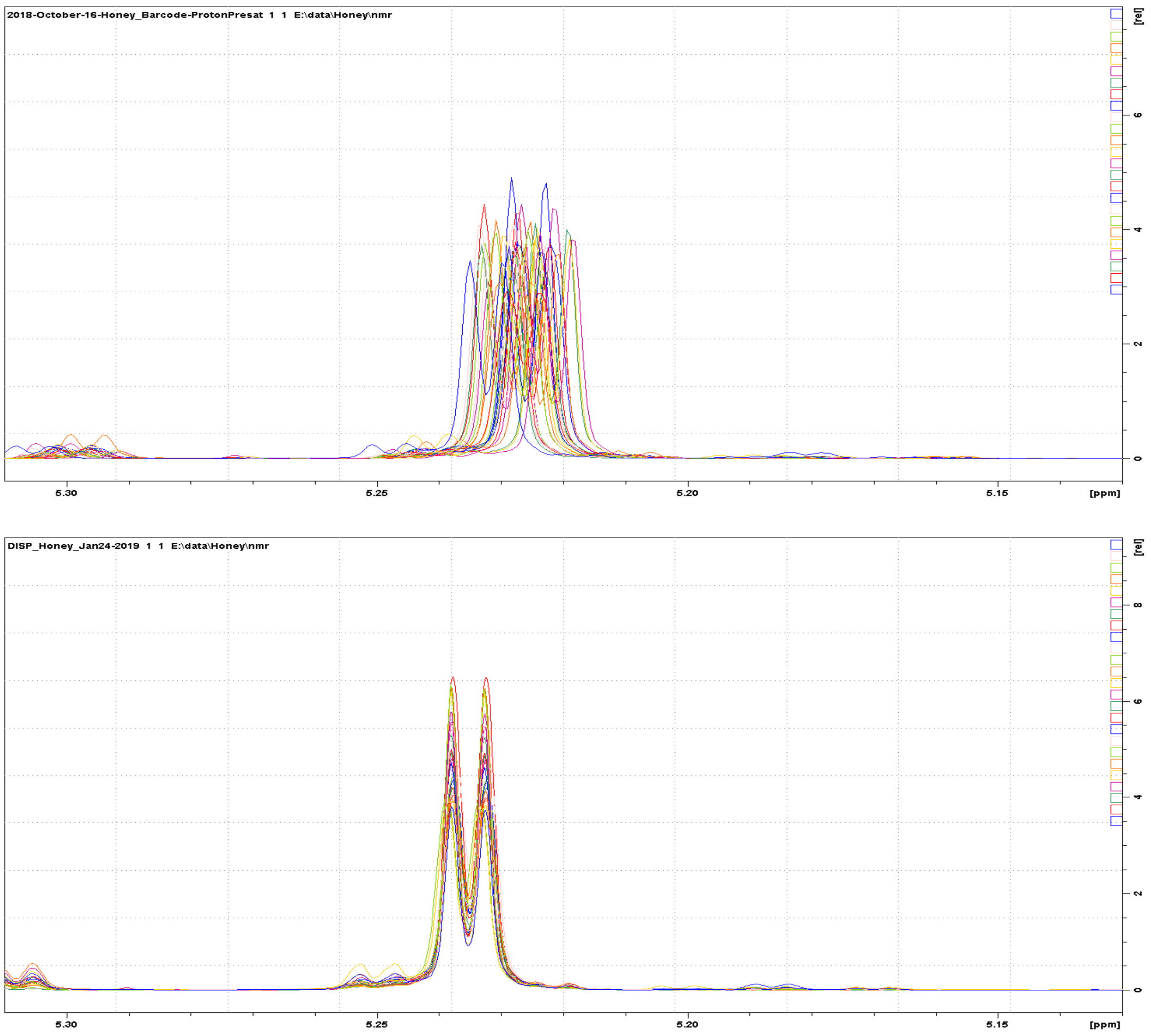

Figure S1. Comparison of 25 different honey samples in D2O (A) and phosphate buffered D2O (B) showing the alpha anomeric proton of glucose. In the D2O samples the deviation of the chemical shift is greater than 8 Hz where in the buffered samples the chemical shift deviation is less than 1 Hz.

Table S1. Integration regions and chemical shift assignments of resonances of the chemical markers quantified in this study. The scaling factor is in relation to the integration of the anomeric protons of sugars where the proton represents only part of the total molecular signal.

	Metabolite	Integration Range		Assignment	Scaling
1	5-HMF	9.475	9.445	9.46	
2	Trigonelline	9.16	9.13	9.14	
3	Formic Acid	8.47	8.44	8.46	
4	Leptosperin	7.462	7.443	7.448	
7	Phenylalanine	7.447	7.4	7.43	
5	Methyl Syringate	7.426	7.412	7.42	
7	Phenylalanine	7.4	7.349	7.421	
7	Phenylalanine	7.349	7.296	7.38	
8	p-Hydroxyphenyllactic Acid	7.273	7.235	7.256	
9	Tyrosine	7.214	7.172	7.198	
8	p-Hydroxyphenyllactic Acid	6.99	6.953	6.97	
9	Tyrosine	6.917	6.882	6.9	
10	Maltose	5.432	5.42	5.426	0.60
11	Sucrose	5.42	5.406	5.412	1.00
12	Kojibiose	5.404	5.392	5.397	0.57
13	Nigerose	5.379	5.362	5.37	0.57
14	Turanose	5.324	5.295	5.308	0.81
15	Trehalose	5.212	5.195	5.204	1.00
16	DHA	4.428	4.412	4.42	
17	Fructose (β-fur H3, β-fur H4, α-fur H3)	4.13	4.098	4.12	
17	Fructose (β-pyr H6)	4.05	4.013	4.03	
17	Fructose (β-pyr H5, α-fur H4)	4.013	3.985	4	
18	Glucose H-4 α,β	3.435	3.38	3.41	
6	Phenyllactic Acid	2.91	2.86	2.88	
19	Citric Acid	2.569	2.519	2.54	
20	Malic Acid	2.7	2.655	2.67	
21	Succinic Acid	2.418	2.404	2.41	
22	Methylglyoxal monohydrate	2.313	2.299	2.31	
23	Proline	2.106	2.049	2.07	
24	Acetate	1.928	1.918	1.92	
25	Proto-quercitol	1.846	1.790	1.82	
26	Alanine	1.492	1.472	1.482	
27	Lactate	1.396	1.369	1.383	
28	Methylglyoxal dihydrate	1.381	1.367	1.376	
29	Threonine	1.344	1.319	1.332	
30	Ethanol	1.202	1.173	1.187	
31	3-Butanediol	1.157	1.132	1.145	
32	Valine	1.056	1.035	1.046	
33	Isoleucine	1.024	1.005	1.015	
32	Valine	1.005	0.983	0.994	
	TMSP	0.095	-0.1	0	

Table S2. Average concentrations of each analyte over all 424 samples.

Analyte	Mean	S.D	Units
5-HMF	35.08	53.44	mg/kg
Trigonelline	13.70	14.29	mg/kg
Formic Acid	60.15	60.94	mg/kg
Leptosperin	27.73	75.58	mg/kg
MeSyringate	61.49	77.33	mg/kg
PLA	40.73	134.68	mg/kg
Phenylalanine	161.80	206.09	mg/kg
HPLA	19.59	77.59	mg/kg
Tyrosine	46.36	63.57	mg/kg
Maltose	1.90	0.97	g/100g
Sucrose	1.13	1.22	g/100g
Kojibiose	0.72	0.30	g/100g
Nigerose	0.47	0.21	g/100g
Turanose	1.34	0.56	g/100g
Trehalose	0.09	0.10	g/100g
DHA	44.89	67.80	mg/kg
Fructose	40.91	2.28	g/100g
Glucose	35.12	2.99	g/100g
Citrate	95.17	178.71	mg/kg
Malic Acid	43.84	142.42	mg/kg
Succinic Acid	38.63	53.73	mg/kg
MGMH	9.56	22.14	mg/kg
Proline	495.53	250.05	mg/kg
Acetate	40.54	31.60	mg/kg
Alanine	36.20	82.49	mg/kg
Lactate	53.24	135.68	mg/kg
MGDH	29.21	41.86	mg/kg
Threonine	158.89	143.50	mg/kg
EtOH	68.23	104.14	mg/kg
1,3-Butanediol	72.49	249.22	mg/kg
Valine	17.69	20.76	mg/kg
Isoleucine	17.60	27.17	mg/kg
Fru+Glu	76.03	2.96	mg/kg
Fru/Glu	1.18	0.15	mg/kg

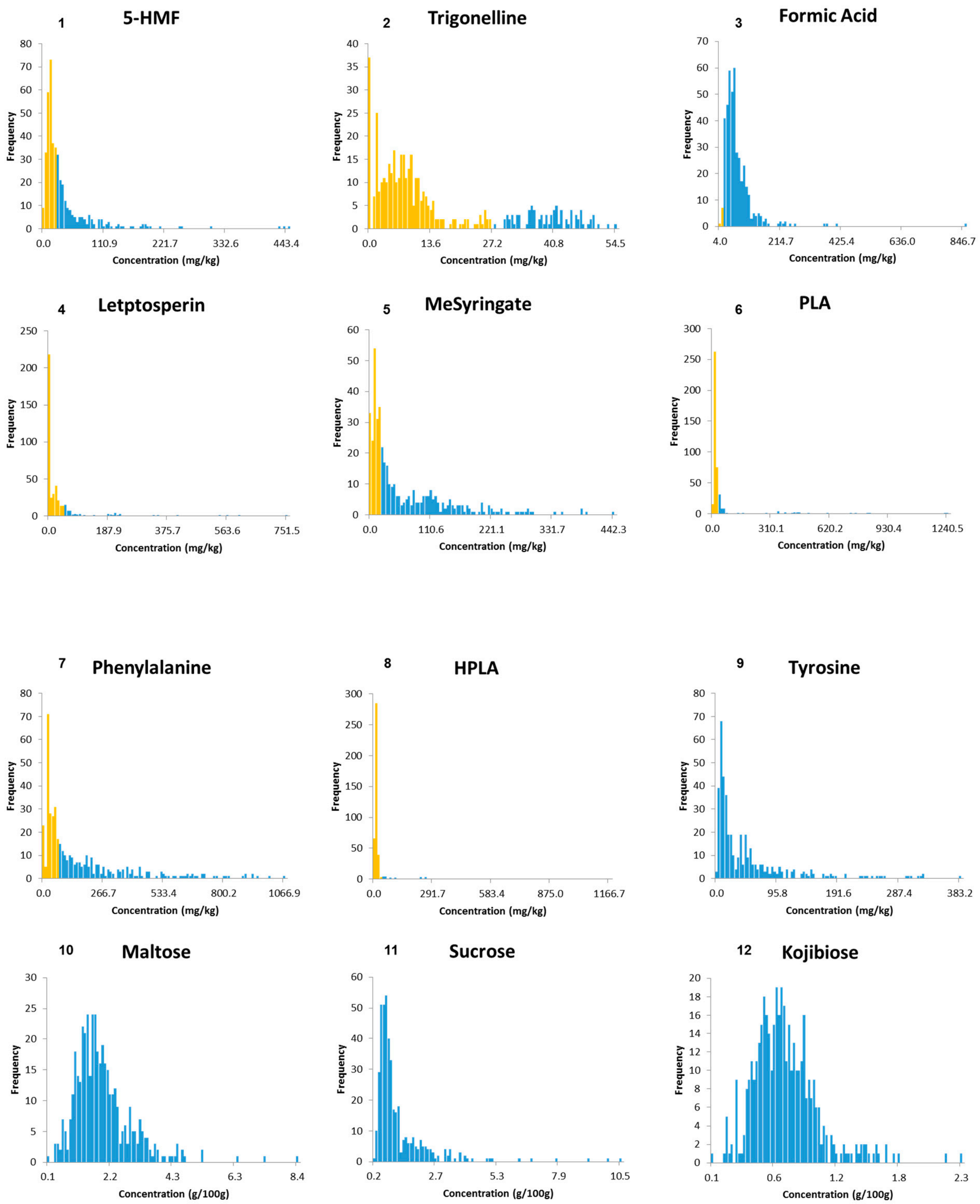

1
5-HMF
Frequency
Concentration (mg/kg)
0.0
110.9
221.7
332.6
443.4
2
Trigonelline
Frequency
Concentration (mg/kg)
0.0
13.6
27.2
40.8
54.5
3
Formic Acid
Frequency
Concentration (mg/kg)
4.0
214.7
425.4
636.0
846.7
4
Letptosperin
Frequency
Concentration (mg/kg)
0.0
187.9
375.7
563.6
751.5
5
MeSyringate
Frequency
Concentration (mg/kg)
0.0
110.6
221.1
331.7
442.3
6
PLA
Frequency
Concentration (mg/kg)
0.0
310.1
620.2
930.4
1240.5
7
Phenylalanine
Frequency
Concentration (mg/kg)
0.0
266.7
533.4
800.2
1066.9
8
HPLA
Frequency
Concentration (mg/kg)
0.0
291.7
583.4
875.0
1166.7
9
Tyrosine
Frequency
Concentration (mg/kg)
0.0
95.8
191.6
287.4
383.2
10
Maltose
Frequency
Concentration (g/100g)
0.1
2.2
4.3
6.3
8.4
11
Sucrose
Frequency
Concentration (g/100g)
0.2
2.7
5.3
7.9
10.5
12
Kojibiose
Frequency
Concentration (g/100g)
0.1
0.6
1.2
1.8
2.3

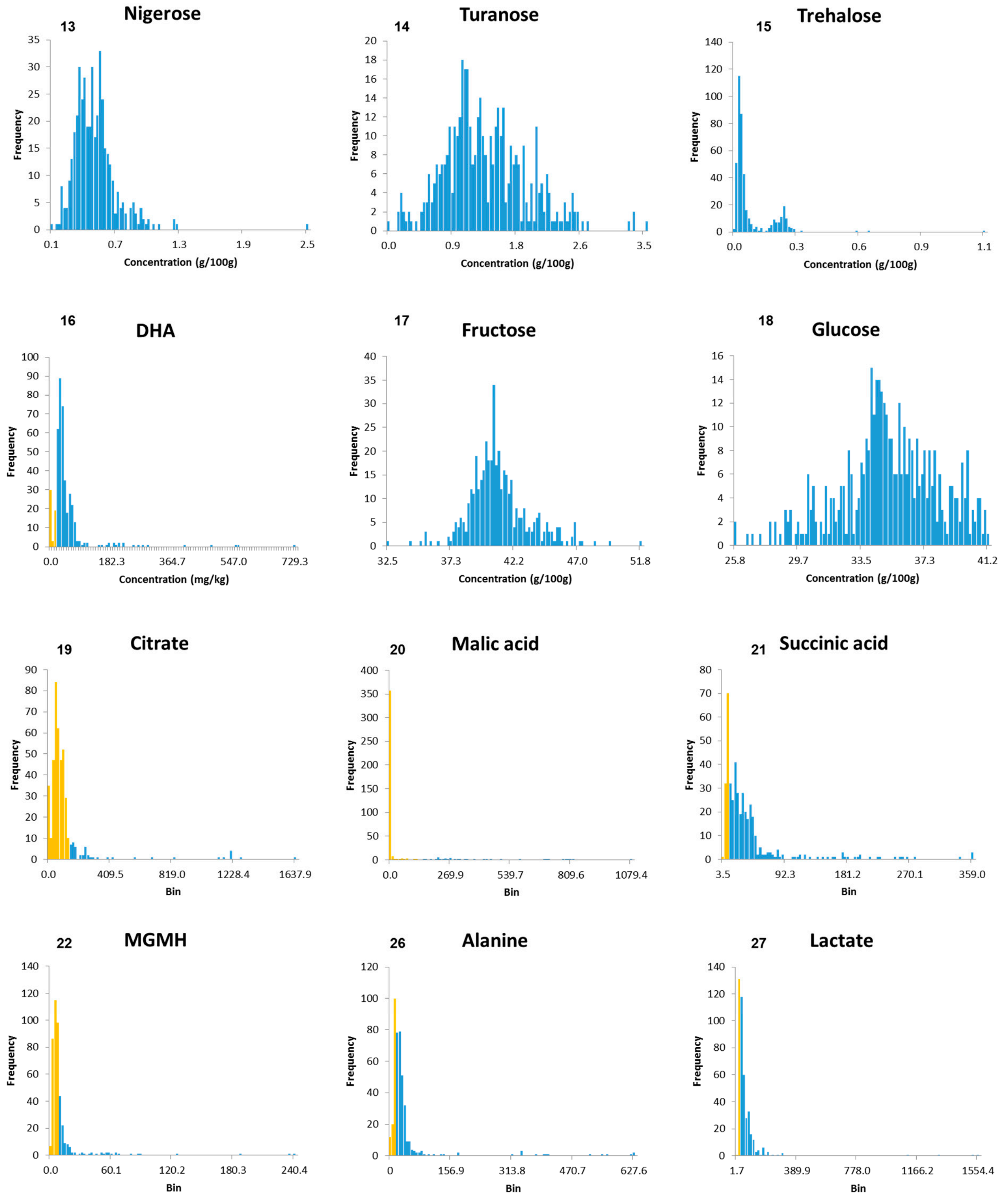

13 Nigerose
Frequency
0.1 0.7 1.3 1.9 2.5
Concentration (g/100g)
14 Turanose
Frequency
0.0 0.9 1.8 2.6 3.5
Concentration (g/100g)
15 Trehalose
Frequency
0.0 0.3 0.6 0.9 1.1
Concentration (g/100g)
16 DHA
Frequency
0.0 182.3 364.7 547.0 729.3
Concentration (mg/kg)
17 Fructose
Frequency
32.5 37.3 42.2 47.0 51.8
Concentration (g/100g)
18 Glucose
Frequency
25.8 29.7 33.5 37.3 41.2
Concentration (g/100g)
19 Citrate
Frequency
0.0 409.5 819.0 1228.4 1637.9
Bin
20 Malic acid
Frequency
0.0 269.9 539.7 809.6 1079.4
Bin
21 Succinic acid
Frequency
3.5 92.3 181.2 270.1 359.0
Bin
22 MGMH
Frequency
0.0 60.1 120.2 180.3 240.4
Bin
26 Alanine
Frequency
0 156.9 313.8 470.7 627.6
Bin
27 Lactate
Frequency
1.7 389.9 778.0 1166.2 1554.4
Bin

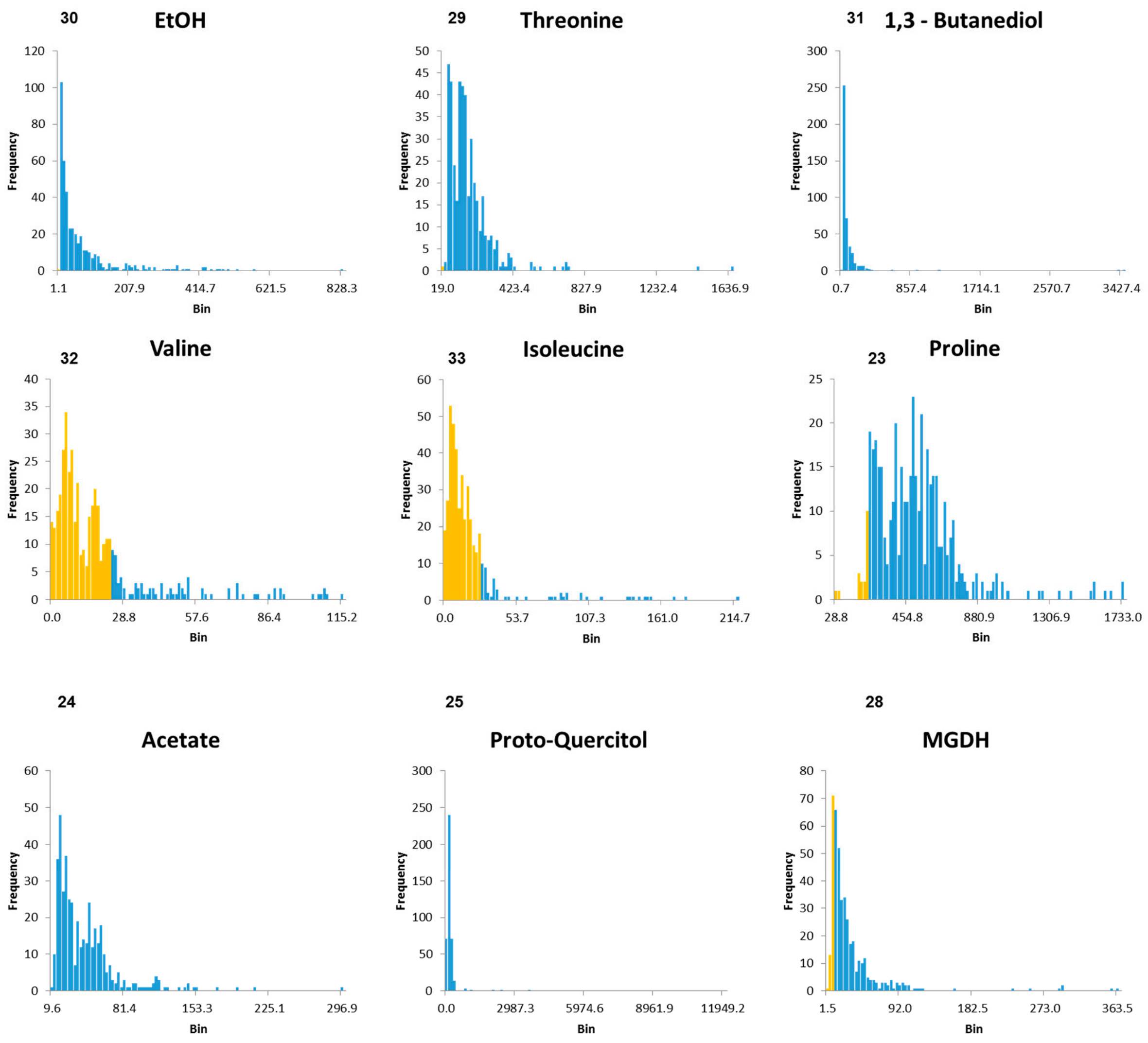

Figure S2. Frequency distributions of the concentrations for each analyte. The selection bin sizes for the histograms is described in the main article.
